# Effects of Methylone Pre-Exposure on Fluoxetine-Induced Conditioned Taste Avoidance in Male and Female Sprague-Dawley Rats

**DOI:** 10.3390/brainsci13040585

**Published:** 2023-03-30

**Authors:** Hayley N. Manke, Kenner C. Rice, Anthony L. Riley

**Affiliations:** 1 Psychopharmacology Laboratory, Center for Neuroscience and Behavior, Department of Neuroscience, American University, 4400 Massachusetts Ave, NW, Washington, DC 20016, USA; 2 Drug Design and Synthesis Section, National Institute on Drug Abuse (NIDA), National Institute on Alcohol Abuse and Alcoholism (NIAAA), Bethesda, MD 20892, USA

**Keywords:** methylone, fluoxetine, conditioned taste avoidance, pre-exposure, 5-HT

## Abstract

Background: Prior work has reported that a drug’s aversive effects (as indexed by taste avoidance conditioning) are attenuated when the pre-exposure and conditioning drugs are the same or different. The latter, otherwise known as cross-drug pre-exposure, is especially interesting as it has been used as a tool to assess mechanisms underlying the aversive effects of drugs. We previously reported that methylone pre-exposure differentially impacted the aversive effects of MDPV and MDMA (MDPV > MDMA), a difference consistent with the dopaminergic mediation of methylone’s aversive effects. To examine the possible role of serotonin (5-HT) in methylone’s aversive effects, the present study assessed the effects of methylone pre-exposure on taste avoidance induced by the 5-HT reuptake inhibitor fluoxetine. Methods: Male and female Sprague-Dawley rats were exposed to 10 mg/kg of methylone every 4th day (for a total of 5 injections) prior to taste avoidance training with 10 mg/kg of fluoxetine. Results: Fluoxetine induced significant taste avoidance (each *p* < 0.05) that was independent of sex. Methylone pre-exposure had no impact on avoidance produced by fluoxetine in either males or females (each *p* > 0.05). Conclusions: Methylone pre-exposure had no impact on fluoxetine-induced avoidance. These findings suggest that it is unlikely that 5-HT mediates the aversive effects of methylone. The implications of the present results for the mechanisms mediating methylone’s aversive effects were discussed. Understanding such mechanisms is important in predictions relevant to drug history and abuse liability as a variety of subject and experiential factors known to affect (reduce) a drug’s aversive effects may increase its use and potential for abuse.

## 1. Introduction

Conditioned taste avoidance (CTA) was first empirically demonstrated in the 1950s in the context of military applications, wherein researchers investigated the impact of toxins on rodent infestations (i.e., bait-shyness; [1]) and the effects of radiation exposure on biological systems [2] (for a history of CTAs, see [3]). In these reports, animals avoided consuming a particular taste or food following its pairing with an aversive agent [4,5,6]. Following these initial demonstrations, subsequent work demonstrated that a wide range of compounds, including various toxins and drugs of abuse, suppress consumption following their pairing with a novel taste (presumably as a function of the compounds’ aversive effects; see [7,8,9]; for lists of compounds producing CTAs, see [10]).

Although CTAs were initially investigated within the context of toxicology and constraints on learning (given that they occurred under conditions that did not generally support learning under more traditional assessments; see [4,5,6]), more recently a large body of research has extended the analysis of taste avoidance learning to assess the impact of a number of subject and experiential factors on its acquisition and display (see [3,10,11]). In this context, one factor that has received considerable attention is drug history (for reviews, see [12,13]). Specifically, animals exposed to a drug prior to its pairing with a novel solution display attenuated taste avoidance compared to animals with no pre-exposure. For instance, in one of the initial demonstrations of the effects of pre-exposure, Berman and Cannon [14] reported that rats exposed to ethanol prior to taste avoidance conditioning displayed attenuated ethanol-induced avoidance relative to non-pre-exposed subjects. Since this report, studies using a range of drugs have demonstrated the attenuating effects of drug pre-exposure [15,16]. Importantly, the attenuating effects of drug pre-exposure have also been observed in cross-drug preparations in which the pre-exposure and conditioning drugs differ ([17,18,19,20]; for a review, see [13]).

Although there is no consensus on the mechanism underlying the attenuating effects of drug history on CTAs, it has been argued that during drug pre-exposure the animal habituates or becomes tolerant to the drug’s aversive effects reducing its ability (and that of other drugs with similar or shared stimulus properties) to condition an avoidance when subsequently paired with a novel taste (see [7,17,18]; for other interpretations of drug pre-exposure, see [21,22]; for a discussion of associative and non-associative interpretations, see [13]). Independent of the basis of drug pre-exposure, each contends that the attenuation produced results from some similarity between the aversive effects of the pre-exposure and conditioning drugs (see [13,18,19,20]).

Given that the cross-drug pre-exposure design utilizes different pre-exposure and conditioning drugs, it allows researchers to evaluate the possible mechanisms underlying the aversive effects of the compounds tested by assessing their shared or common stimulus properties. In an early demonstration of the cross-drug pre-exposure effect, De Beun et al. [17] examined cross-familiarization in this procedure to reveal stimulus similarities between the selective 5-HT_1A_ agonist 8-OH-DPAT and a variety of serotonergic and non-serotonergic drugs. The results of the work revealed that pre-exposure to 8-OH-DPAT and other agonists at the 5-HT_1A_ receptor (either on the receptor or functional level) attenuated 8-OH-DPAT-induced avoidance. Dopamine (DA) antagonists, adrenergic agonists, 5-HT_1A_ antagonists, and drugs acting at other 5-HT_1_ receptor subtypes were ineffective in attenuating avoidance produced by 8-OH-DPAT. Taken together, these results support the ability of the cross-drug design to reveal similarities between the aversive effects of various compounds (although such cross-generalization may be drug-dependent) and the mechanisms that may underlie these effects. The ability to understand these similarities (or differences) and the mechanisms underlying these effects are important as they may facilitate predictions of abuse liability. Specifically, if drugs share stimulus properties, it would be expected that they will interact in such a way as to abate each other’s aversive effects (as a function of drug history) and to increase their use.

We have recently extended the analysis of cross-drug pre-exposure to a relatively new class of psychoactive substances, i.e., the synthetic cathinones (aka “bath salts”; [23,24,25]). For example, Woloshchuk et al. [25] reported that pre-exposure to MDPV (a first-generation synthetic cathinone) attenuated avoidance induced by itself as well as that induced by the psychostimulant cocaine. MDPV pre-exposure had no effect on avoidance induced by the emetic LiCl. Interestingly, MDPV pre-exposure had a greater attenuating effect on itself than that of cocaine. This difference in the degree of attenuation is expected given that although cocaine and MDPV share similarities in their mechanism of action (both act as monoamine reuptake inhibitors), they differ in their potencies at the monoamine transporters (MDPV > cocaine; [26,27]). Recently, Manke et al. [23] reported that rats exposed to methylone (another first-generation synthetic cathinone) prior to taste avoidance conditioning, with either MDPV or MDMA [23], displayed attenuated avoidance with the degree of attenuation dependent on the conditioning drug (MDPV > MDMA). Methylone is mixed in its neurochemical activity, blocking the reuptake of the monoamines as well as serving as a substrate releaser primarily for 5-HT. The fact that methylone pre-exposure attenuated avoidance induced by MDPV (a selective monoamine reuptake inhibitor) more than MDMA (a 5-HT substrate releaser; [26,27]) suggests that the aversive effects of methylone may be mediated more by its effects on the catecholamines (DA and NE) than 5-HT. Consequently, its effects on MDMA (and other compounds with predominant serotonergic activity) would be minimal.

To address this issue, the present study examined the effects of methylone pre-exposure on fluoxetine-induced taste avoidance. Fluoxetine is generally described as a selective 5-HT reuptake inhibitor (i.e., SSRI), although it also has been reported to have weak inhibitory activity at NET, disinhibit NE activity, act as an antagonist at 5-HT_2C_ receptors, and inhibit CYP2D6 [28]. Fluoxetine has been reported to induce significant taste avoidance (see [20,29,30]), which is attenuated by its own pre-exposure [20,31]. Further, although fluoxetine and MDMA work by different mechanisms to elevate 5-HT levels (selective 5-HT reuptake inhibitor and 5-HT substrate releaser, respectively), these two compounds have been reported to interact in a variety of behavioral preparations. For instance, fluoxetine pre-exposure attenuates the positive subjective effects of MDMA [32]. Fluoxetine pre-exposure also attenuates MDMA-induced anxiety and depression [33]. Recently, Bowman et al. [34] reported that fluoxetine pre-exposure attenuated MDMA-induced conditioned taste avoidance in males but not females. Collectively, these studies suggest an overlap of MDMA and fluoxetine neurochemical activity, specifically 5-HT. Given such an overlap and our prior work implicating DA (and possibly NE) in methylone’s aversive effects (see above), it is expected that methylone pre-exposure is unlikely to impact fluoxetine-induced taste avoidance. To address this issue, methylone was administered every fourth day for a total of five injections prior to taste avoidance conditioning with fluoxetine. The present assessment was performed in both male and female Sprague-Dawley rats due to the reported sex differences with fluoxetine ([34,35,36]; for other discussions of sex as a biological variable in taste avoidance conditioning as well as in drug use and abuse; see [37,38]).

## 2. Methods

### 2.1. Subjects

Adult male (*n* = 33) and female (*n* = 32) Sprague-Dawley (SD) rats were bred within the American University animal research facility and matured undisturbed until the start of testing (approximately postnatal day (PND) 82). Subjects were weighed daily for 7 days (PND 82-88) to index health and reduce handling stress before the start of testing, at which point males weighed between 294 and 433 g (mean = 371; SEM = 5.73) and females weighed between 197 and 294 g (mean = 245; SEM = 3.47). The procedures utilized in the present study adhered to the Guidelines for the Care and Use of Laboratory Animals [39] and the Guidelines for the Care and Use of Mammals in Neuroscience and Behavioral Research [40] and were approved (under protocol 18-12 and 20-02) by the Institutional Animal Care and Use Committee at American University.

### 2.2. Drugs and Solutions

Racemic methylone and fluoxetine used in the present study were dissolved in isotonic saline (0.9%) and both were injected intraperitoneally (IP) at 10 mg/kg. Methylone was generously synthesized and provided by the drug design and synthesis section (MTMDB, NIDA, and NIAAA) and fluoxetine hydrochloride was synthesized by Spectrum Chemical MFG. Corp. All drug and vehicle solutions were prepared daily and subsequently passed through a 0.2 um filter, prior to injection, to remove any potential particulates. Saccharin (sodium saccharin, Acros Organics, NJ, USA) was prepared as a 1 g/L (0.1%) solution in tap water.

### 2.3. Apparatus

Rats of the same sex were socially housed (2–3 per cage) in OptiRat Plus cages (38.9 cm × 56.9 cm × 26.2 cm; 1181 cm^2^). Animal housing rooms were maintained on a 12 h light/dark cycle (lights on between 0800 and 2000 h) at 23 °C with the humidity level kept between 30–70%. The experimental procedures took place during the lights-on phase of the cycle. Food and water were provided ad libitum unless stated otherwise. For fluid consumption during conditioned taste avoidance training and testing (see below), subjects were placed in individual hanging, stainless-steel wire-mesh test cages (24.3 cm × 19 cm × 18 cm) in which graduated Nalgene tubes were placed on the front of the individual wire-mesh cage for fluid presentation.

### 2.4. Procedure

#### 2.4.1. Phase 1: Habituation

**Figure 1:**
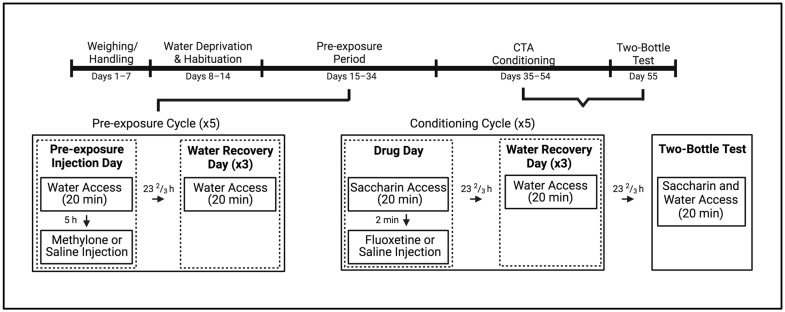
Experimental timeline for subjects pre-exposed to methylone or saline prior to undergoing taste avoidance conditioning with fluoxetine or saline. Created with BioRender.com.

Beginning 24 h prior to the start of water habituation (~PND 89; see Figure 1 for an experimental timeline), male and female SD rats were deprived of water and on the following day (PND 90) were given 20 min access to tap water, presented in 50 mL Nalgene tubes that were placed on the individual wire-mesh testing cages. Subsequent to fluid access, animals were returned to their home cages. This procedure was repeated for 6 days to allow water consumption to stabilize, such that all subjects approached the Nalgene tubes within 2 sec and did not increase or decrease their average volume of water by more than 2 mL for 3 consecutive days. Fluid consumption was calculated by taking the difference between pre- and post-consumption volumes.

#### 2.4.2. Phase 2: Pre-Exposure

On the first day of pre-exposure, all subjects were given 20 min access to tap water and the difference between pre- and post-consumption values was taken. Male and female subjects were assigned to two groups, such that water consumption was comparable. Approximately 5 h later, each subject was injected with either methylone (10 mg/kg) or equivolume saline (*n* = 16–17 per group for males and females). The pre-exposure dose of methylone used here was based on prior research showing that this dose conditioned intermediate taste avoidance [41] and attenuated avoidance induced by MDPV and MDMA [23]. After injections were administered, animals were returned to their home cages. For the next 3 days, the animals were given 20 min access to tap water and did not receive any injections. This cycle of one pre-exposure injection followed by 3 water days was repeated for a total of 5 cycles over the course of 20 days.

#### 2.4.3. Phase 3: Conditioned Taste Avoidance (CTA)

On the 1st day of conditioning, all subjects were placed in the testing cages and presented with a novel saccharin solution for 20 min. Based on saccharin consumption, both male and female subjects from the two pre-exposure conditions were, then, assigned to one of two groups and injected with either the saline vehicle or 10 mg/kg fluoxetine, immediately following saccharin access. This resulted in a total of four groups, i.e., vehicle–vehicle, vehicle–fluoxetine, methylone–vehicle, and methylone–fluoxetine (*n* = 8–9 per group for both males and females), where the first component of the group name indicates the pre-exposure condition of the subject (vehicle or methylone) and the latter represents the conditioning injection (vehicle or fluoxetine). The doses utilized for CTA training and testing were based on previous work demonstrating that 10 mg/kg fluoxetine yielded intermediate taste avoidance [20]. For the next 3 days (days 2–4), all subjects were given access to water for 20 min in the test cages but were not injected. This four-day conditioning cycle was repeated four additional times (total of five cycles). As above, saccharin and water consumption were evaluated by the difference between pre- and post-consumption values. The day after the last conditioning cycle (day 21), subjects were placed in the testing cages and given simultaneous access to saccharin and water in a final two-bottle test, with no injections following fluid access. The present study examined the two-bottle assessment given its increased sensitivity in detecting effects that may not be evident during the one-bottle trials [42,43,44]. In this assessment, one bottle was presented (saccharin or water) on either the left or right front of the testing cage, and immediately after the subject sampled the first bottle, it was removed, and the second bottle was presented on the opposite side of the cage. Again, after it was sampled, the second bottle was removed and then both bottles were presented concurrently on their respective sides on the front of the cage. The order of presentation and side placement were counterbalanced across subjects, and consumption of both saccharin and water was recorded as differences in pre- and post-consumption values. Following the two-bottle test, animals were returned to their home cages with ad libitum water access.

### 2.5. Statistical Analysis

The number of subjects in the present study was determined via power analysis that indicated that *n* ≥ 7 was appropriate to detect significant differences in the anticipated effect sizes, while α = 0.05 and power (1 − β) = 0.8.

The impact of methylone pre-exposure on bodyweight and fluid consumption was assessed using a 2 × 2 × 5 mixed model ANOVA with the between-subjects factor of pre-exposure drug and sex and the within-subjects factor of injection day (1–5) for each measure.

The effects of methylone pre-exposure on fluoxetine-induced CTA over trials (and if these effects varied by sex) were analyzed using a 2 × 2 × 2 × 5 mixed model ANOVA with the between-subject factors of pre-exposure drug (methylone or vehicle), conditioning drug (fluoxetine or vehicle) and sex (male or female) and the within-subjects factor of the trials (1–5). In the case of a significant four-way interaction, simple effects of the trials at each pre-exposure drug, conditioning drug, and sex (multivariate analysis) and the effects of pre-exposure drug at each conditioning drug, trial, and sex (univariate analysis) were assessed followed by Bonferroni-adjusted multiple comparisons. To assess differences in the percentage, saccharin was consumed during the two-bottle test, data were analyzed using a two-way ANOVA with the same between-subjects factors. In the case of a significant two-way interaction, the effects of pre-exposure drug and conditioning drug (univariate analysis) were assessed and followed by Bonferroni-adjusted multiple comparisons. Statistical significance was set at *p* ≤ 0.05.

## 3. Results

### 3.1. Bodyweight and Fluid Consumption over Pre-Exposure

**Figure 2:**
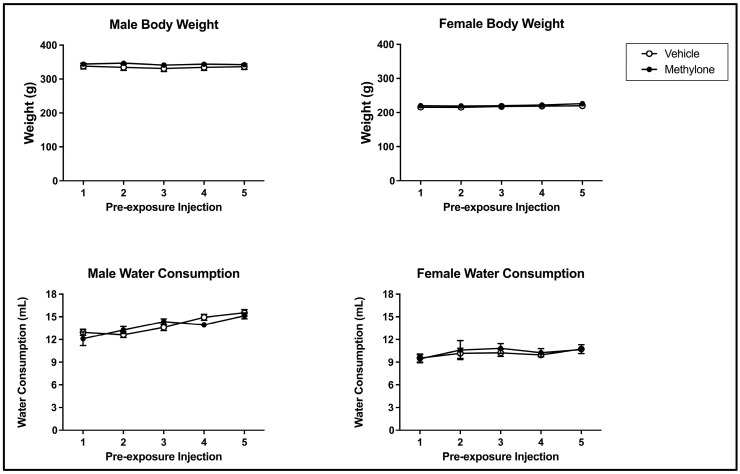
Mean (+/− SEM) bodyweight (g; **top**) and water consumption (mL; **bottom**) of male (**left**) and female (**right**) subjects injected with saline (vehicle) or 10 mg/kg of methylone (*n* = 16–17 per group for both males and females) on pre-exposure days.

Methylone pre-exposure did not impact bodyweight across the pre-exposure phase of the present study. The 2 × 2 × 5 mixed model ANOVA on bodyweight over the course of the pre-exposure phase revealed a main effect of sex (F(1, 61) = 489.487, *p* < 0.001) but not of injection day (F(4, 244) = 2.372, *p* = 0.0.053) or pre-exposure drug (F(1, 61) = 1.467, *p* = 0.230) (see Figure 2; top panels). There was a significant interaction between injection day and sex (F(4, 244) = 3.407, *p* = 0.010) but not injection day x pre-exposure drug (F(4, 244) = 0.429, *p* = 0.788), injection day x pre-exposure drug x sex (F(4, 244) = 1.240, *p* = 0.294) or pre-exposure drug x sex (F(1, 61) = 0.170, *p* = 0.681). The injection day x sex interaction reflects the changes in bodyweight across the pre-exposure differences within each sex. For males, bodyweight significantly increased from injection days 2 to 3 and injection days 3 to 4. Additionally, the bodyweight on injection day 3 significantly differed from those on injection day 5. For females, bodyweight on injection day 5 was significantly higher than that on injection days 2, 3, and 4 (but these intervening days did not differ from each other).

There was also no impact of methylone pre-exposure on water consumption across pre-exposure. The 2 × 2 × 5 mixed model ANOVA on water consumption over the course of pre-exposure revealed that there was a main effect from sex (F(1, 61) = 102.830, *p* < 0.001) and injection day (F(4, 244) = 9.404, *p* < 0.001) but not of pre-exposure drug (F(1, 61) = 0.003, *p* = 0.955) (see Figure 2; bottom panels). There was a significant interaction between injection day and sex (F(4, 244) = 3.226, *p* = 0.013) but not injection day x pre-exposure drug (F(4, 244) = 1.168, *p* = 0.326), injection day x pre-exposure drug x sex (F(4, 244) = 0.422, *p* = 0.793) or pre-exposure drug x sex (F(1, 61) = 0.307, *p* = 0.581). The injection day x sex interaction reflects that the changes in water consumption across pre-exposure differed within each sex. For males, water consumption significantly increased from injection days 1 to 4 and injection days 1 to 5. Water consumption for males was also significantly higher on injection day 5 compared to injection days 2 and 3. For females, water consumption did not differ across injection days (each *p* > 0.05).

### 3.2. Conditioned Taste Avoidance

**Figure 3:**
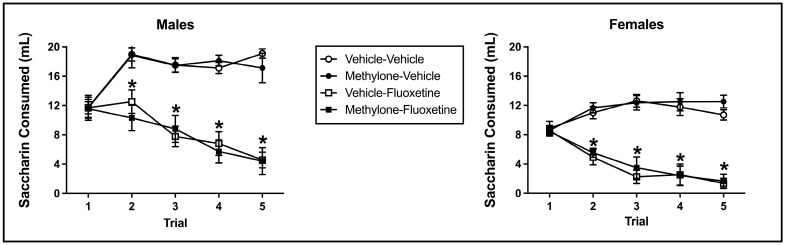
Mean (+/− SEM) saccharin consumption (mL) during taste avoidance conditioning by male (**left**) and female (**right**) subjects injected with saline (vehicle) or methylone during pre-exposure and conditioned with vehicle or 10 mg/kg of fluoxetine (*n* = 8–9 per group for both males and females). * Subjects conditioned with fluoxetine (regardless of pre-exposure condition) significantly differed from controls.

Fluoxetine induced significant taste avoidance that was unaffected by methylone pre-exposure or sex. The mixed model ANOVA on saccharin consumption over conditioned taste avoidance training revealed a main effect from the trial (F(4, 228) = 11.213, *p* < 0.001), conditioning drug (F(1, 57) = 154.748, *p* < 0.001), and sex (F(1, 57) = 61.953, *p* < 0.001) (see Figure 3). Additionally, there was a significant interaction between the trial and conditioning drug (F(4, 228) = 56.339, *p* < 0.001) and trial x sex (F(4, 228) = 4.949, *p* = 0.001). There was no significant main effect of pre-exposure drug (F(1, 57) = 0.004, *p* = 0.951) or trial x pre-exposure drug (F(4, 228) = 0.224, *p* = 0.925), trial x pre-exposure drug x conditioning drug (F(4, 228) = 0.792, *p* = 0.531), trial x pre-exposure drug x sex (F(4, 228) = 0.720, *p* = 0.579), trial x conditioning drug x sex (F(4, 228) = 1.659, *p* = 0.160), trial x pre-exposure drug x conditioning drug x sex (F(4, 228) = 0.696, *p* = 0.596), pre-exposure drug x conditioning drug (F(1, 57) = 0.029, *p* = 0.866), pre-exposure drug x sex (F(1, 57) = 0.448, *p* = 0.506), conditioning drug x sex (F(1, 57) = 0.934, *p* = 0.338), or pre-exposure drug x conditioning drug x sex (F(1, 57) = 0.002, *p* = 0.966) interactions.

In relation to the significant trial x conditioning drug interaction, beginning on trial 2 and continuing through trial 5, subjects injected with fluoxetine (collapsed across sex and pre-exposure drug) drank significantly less than controls (each *p* < 0.001), indicative of fluoxetine-induced taste avoidance (see Figure 3). Fluoxetine-injected animals also significantly decreased their saccharin consumption across trials (i.e., trial 1 to trial 5; *p* < 0.001). The main effect of sex and the trial x sex interaction (collapsed across conditioning drug and pre-exposure drug) reflect the fact that males presented a higher absolute saccharin consumption on all trials relative to female subjects (each *p* < 0.001).

### 3.3. Two-Bottle Avoidance Test

**Figure 4:**
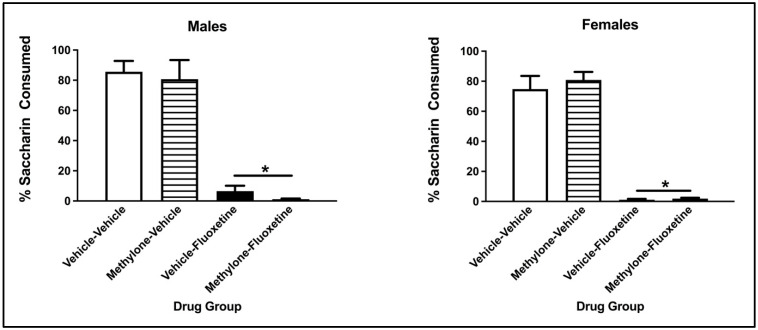
Mean (+/− SEM) percent saccharin consumed by male (**left**) and female (**right**) subjects injected with saline (vehicle) or methylone during pre-exposure and conditioned with vehicle or 10 mg/kg of fluoxetine (*n* = 8–9 per group for both males and females). * Subjects conditioned with fluoxetine (regardless of pre-exposure condition) significantly differed from controls.

The three-way ANOVA on the percentage of saccharin consumption during the two-bottle test revealed a significant main effect from the conditioning drug (F(1, 57) = 298.317, *p* < 0.001) but not the pre-exposure drug (F(1, 57) = 0.040, *p* = 0.841) or Sex (F(1, 57) = 0.715, *p* = 0.401). There was no significant interaction between the conditioning drug and pre-exposure drug (F(1, 57) = 0.096, *p* = 0.757), sex x pre-exposure drug (F(1, 57) = 0.908, *p* = 0.345), sex x conditioning drug (F(1, 57) = 0.101, *p* = 0.751) or sex x conditioning drug x pre-exposure drug (F(1, 57) = 0.080, *p* = 0.779). Collapsed across sex and pre-exposure drug, males and females conditioned with fluoxetine drank a significantly lower percentage of saccharin compared to vehicle animals (*p* < 0.001; see Figure 4).

## 4. General Discussion

Previous work has demonstrated that the aversive effects of a drug are attenuated following its exposure prior to taste avoidance conditioning. Such a reduction in the aversive effects as a function of pre-exposure has also been reported when the pre-exposure and conditioning drugs are different. The latter is especially interesting given it may be utilized to uncover mechanisms underlying the aversive effects of drugs. In this context, we previously reported that methylone pre-exposure differentially impacted the aversive effects of MDPV and MDMA (MDPV > MDMA; [23]; see above), a difference consistent with dopaminergic mediation of methylone’s aversive effects. To further investigate this possibility, the present study examined the effect of methylone pre-exposure on fluoxetine-induced taste avoidance, a drug with primarily serotonergic actions as a selective 5-HT reuptake inhibitor. As described above, fluoxetine induced significant taste avoidance in males and females, which was not impacted by methylone pre-exposure.

The ability of fluoxetine to produce aversive effects (indexed by CTA) is consistent with prior work that reported significant fluoxetine-induced taste avoidance (dose-dependent) in male rats [20,29,30]. While prior work has reported the aversive effects of fluoxetine, the present study is the first to examine sex differences in such effects (for reviews discussing sex as a biological variable, see [37,38]). The present assessment was made given that sex-dependent taste avoidance has been reported for a variety of drugs (with the presence and direction of sex differences a function of the drug; for reviews, see [38,45]) as well as the fact that other work with fluoxetine (in males and females) has reported sex differences in other behavioral endpoints (see [46,47]). In the present experiment, males and females did not differ in relation to fluoxetine-induced avoidance (over-conditioning or in the two-bottle assessment). Although no differences in fluoxetine-induced avoidance were observed here relating to gender, further investigations are needed as only one dose was assessed in the present study and studies with other drugs have shown that sex differences can be dose-dependent [48,49,50].

While fluoxetine produced significant taste avoidance, the avoidance was not impacted by methylone pre-exposure (see above). Beginning on trial 2, animals injected with fluoxetine during training, regardless of pre-exposure condition, consumed significantly less saccharin than animals conditioned with the vehicle, and the similarities between methylone and saline pre-exposed animals were maintained over the remainder of the conditioning and in the final two-bottle test. While a pre-exposure effect was not found in the present work, it is important to extend the present analysis as only one pre-exposure and conditioning dose was administered and these effects are often dose-dependent (for pre-exposure, see [14,17,18,51]; for CTA, see [2,52]). Although only one pre-exposure and one conditioning dose were used, the doses chosen were based on prior work (see above) demonstrating that the dose of methylone used during pre-exposure attenuates avoidance induced by other compounds [23] and the dose of fluoxetine used in conditioning can be attenuated by prior exposure to other drugs [20,31].

These results support the position that methylone and fluoxetine do not share stimulus properties and that avoidance induced by these two drugs differs. From prior work, avoidance induced by methylone appears to be mediated by DA as methylone significantly impacted avoidance induced by MDPV, a monoamine reuptake inhibitor with primary action on DA, and less attenuation on that induced by MDMA, which is a substrate releaser for 5-HT and an inhibitor of DAT and NET. Unlike MDMA, fluoxetine is a selective 5-HT reuptake inhibitor with little effect on DA or NE. Consistent with its actions on 5-HT, Berendsen and Broekkamp [31] reported that mice pre-exposed to the 5-HT_2c_ agonist MK 212 displayed attenuated fluoxetine-induced CTA. Interestingly, MK 212 only partially attenuated the aversive effects of the 5-HT_7_/5-HT_1A_ receptor agonist 8-OH-DPAT (and no attenuation on the moderately selective *5-HT*_2A_ agonist DOI), suggesting that fluoxetine’s stimulus effects may be mediated primarily by 5-HT activity at the 5-HT_2C_ receptor. Similar work assessing the effects of pre-exposure to other drugs on fluoxetine-induced avoidance is limited (see [20]; for a review, see [53]); however, drug discrimination learning (DDL) procedures that are also used to determine shared stimulus properties (albeit not necessarily those associated with the drugs’ aversive effects; see [54]) support the fact that fluoxetine’s stimulus effects are primarily mediated by its actions on 5-HT. Establishing discriminative control when fluoxetine is used as a training drug in the DDL procedure has proven difficult, likely due to its long half-life (see [55]; though see [56] for a discussion of other SSRIs that have been successfully demonstrated in this design); however, several studies have demonstrated that fluoxetine generalizes to serotonergic compounds ([57,58]; though see [59]) and potentiates the discriminability of several compounds with serotonergic activity [58,60,61]. Further, drugs with primarily dopaminergic activity do not typically generalize to fluoxetine [60,62], indicating that fluoxetine’s stimulus effects are not likely mediated by DA (though see [20]). Instead, cocaine cross-generalizes with other dopaminergic compounds [60,63], effects that can be blocked by DA antagonists (see [64,65]).

In relation to the effects of methylone pre-exposure, both males and females displayed comparable fluoxetine-induced avoidance, which was unaffected by methylone. Research on drug pre-exposure in taste avoidance learning is limited despite the fact that sex differences in taste avoidance learning, itself, have been well characterized (see above). In the few studies that have addressed sex differences in the effects of drug preexposure, the results are somewhat mixed. For example, pre-exposure to either THC [51] or nicotine [66] attenuated avoidance in both male and female rats with no sex differences in this attenuation. Ethanol pre-exposure, however, attenuated avoidance in males, but not females ([49]; see also [67]). Although no studies have addressed sex differences in the effects of drug history on fluoxetine-induced avoidance, Bowman et al. [34] recently reported that pre-exposure to fluoxetine attenuated avoidance induced by MDMA only in males (females displayed MDMA-induced avoidance at levels similar to animals pre-exposed to vehicle). The fact that there was no evidence of the effects of methylone history on fluoxetine-induced avoidance in females adds to the list of such assessments and supports the position that the effects of drug history are drug- and sex-dependent.

The present study used the cross-drug pre-exposure design to evaluate the effects of methylone pre-exposure on taste avoidance produced by fluoxetine. The basis for employing such a design is that any attenuating effects observed are a function of similarities in the aversive effects of the pre-exposure and conditioning drug (e.g., methylone and fluoxetine; see above). In this context, the absence of a pre-exposure effect suggests that the aversive effects of methylone and fluoxetine are likely mediated by different mechanisms. It should be noted, however, that although cross-drug pre-exposure has been used to observe similarities (or differences) in the aversive effects between compounds, the nature of these effects has not been identified. This presents an important caveat in the use of the cross-drug pre-exposure design in assessing common mechanisms, such as identifying or isolating specific effects mediating the aversions induced by drugs, especially in cases where drugs have multiple neurochemical actions. The presence or absence of a pre-exposure effect could be due to similarities or differences at the neurochemical level or at some other level more downstream, e.g., sickness, stress, or disruption in homeostasis (for a related discussion, see [68]). Importantly, this does not argue against the use of the cross-drug design to assess common mechanisms. Rather, the conclusions we may draw in this design implicate a common aversive state and not necessarily the specifics of the state or how it is generated. Interpretations of the results in this design must be cautiously made and examined in comparison to work from other designs assessing the basis of avoidance learning (e.g., via KI/KO manipulations or selective pharmacological agonists/antagonists; see [69,70,71]).

## 5. Scope

Rats conditioned with fluoxetine displayed significant conditioned taste avoidance independent of sex and pre-exposure conditions. Given fluoxetine’s selectivity for 5-HT (as indexed by its neurochemical action and effects in drug discrimination learning and other cross-drug pre-exposure designs; see above), the present results suggest that 5-HT does not mediate the aversive effects of methylone. The importance of assessing the impact of drug history on the aversive effects of various compounds is related to the abuse potential of a drug, given that the likelihood of its use and abuse is a function of the balance of its rewarding and aversive effects (for discussions, see [10,69]). Understanding a drug’s aversive effects (and how these effects are similar to or different from other drugs) may give insight into predictions relevant to drug history and abuse liability since the subject and experiential factors known to impact (reduce) a drug’s aversive effects may increase its use and potential for abuse.

## 6. Limitations

Although the data reported here suggest it is unlikely that 5-HT plays a role in the aversive effects of methylone, the present work does not address the possible role that other neurotransmitters (e.g., DA and NE) may play in its aversive effects or the contributions of such effects relative to 5-HT. It is clear that data from various studies (e.g., DDL, cross-drug pre-exposure, pharmacological manipulations in the CTA design) in other neurotransmitter systems are needed to make these types of determinations. The convergence of the results from each of these behavioral assays may be necessary to determine the mechanisms underlying methylone’s aversive properties. Further, the present study utilized a single dose for pre-exposure and conditioning and given that both effects can be dose-dependent, it is important to extend the analysis to other doses.

## Data Availability

The data presented in this study are available upon request to the corresponding author.
